# Identification and prediction of difficult-to-treat rheumatoid arthritis patients in structured and unstructured routine care data: results from a hackathon

**DOI:** 10.1186/s13075-021-02560-5

**Published:** 2021-07-08

**Authors:** Marianne A. Messelink, Nadia M. T. Roodenrijs, Bram van Es, Cornelia A. R. Hulsbergen-Veelken, Sebastiaan Jong, L. Malin Overmars, Leon C. Reteig, Sander C. Tan, Tjebbe Tauber, Jacob M. van Laar, Paco M. J. Welsing, Saskia Haitjema

**Affiliations:** 1 Department of Rheumatology and Clinical Immunology, University Medical Center Utrecht, Utrecht University, Heidelberglaan 100, 3508 GA Utrecht, The Netherlands; 2 Central Diagnostic Laboratory, University Medical Center Utrecht, Utrecht University, Heidelberglaan 100, 3508 GA Utrecht, The Netherlands; 3 MedxAI, Theophile de Bockstraat 77-1, 1058VA Amsterdam, The Netherlands; 4 Department of Information Technology, University Medical Center Utrecht, Utrecht University, Utrecht, The Netherlands

**Keywords:** Difficult-to-treat rheumatoid arthritis, Routine care data, Applied data analytics in medicine, Machine learning

## Abstract

**Background:**

The new concept of difficult-to-treat rheumatoid arthritis (D2T RA) refers to RA patients who remain symptomatic after several lines of treatment, resulting in a high patient and economic burden. During a hackathon, we aimed to identify and predict D2T RA patients in structured and unstructured routine care data.

**Methods:**

Routine care data of 1873 RA patients were extracted from the Utrecht Patient Oriented Database. Data from a previous cross-sectional study, in which 152 RA patients were clinically classified as either D2T or non-D2T, served as a validation set. Machine learning techniques, text mining, and feature importance analyses were performed to identify and predict D2T RA patients based on structured and unstructured routine care data.

**Results:**

We identified 123 potentially new D2T RA patients by applying the D2T RA definition in structured and unstructured routine care data. Additionally, we developed a D2T RA identification model derived from a feature importance analysis of all available structured data (AUC-ROC 0.88 (95% CI 0.82–0.94)), and we demonstrated the potential of longitudinal hematological data to differentiate D2T from non-D2T RA patients using supervised dimension reduction. Lastly, using data up to the time of starting the first biological treatment, we predicted future development of D2TRA (AUC-ROC 0.73 (95% CI 0.71–0.75)).

**Conclusions:**

During this hackathon, we have demonstrated the potential of different techniques for the identification and prediction of D2T RA patients in structured as well as unstructured routine care data. The results are promising and should be optimized and validated in future research.

**Supplementary Information:**

The online version contains supplementary material available at 10.1186/s13075-021-02560-5.

## Background

The treatment for rheumatoid arthritis (RA) has substantially improved over the past decades, enabling many patients to reach and maintain a state of low disease activity or even remission [1]. However, even when following current management recommendations, there is still a subgroup of patients that remains symptomatic after treatment with several (biological and/or targeted synthetic) disease-modifying antirheumatic drugs ((b/ts)DMARDs) [1–3]. These patients are referred to as having “difficult-to-treat (D2T)” RA. Depending on the definition used, this disease state is estimated to affect 5 to 20% of all RA patients [2–4]. D2T RA is likely the subgroup of RA patients with the highest medical need [5–7]. Identifying and optimizing treatment could thus have great clinical impact for individual patients as well as for the sustainability of the healthcare system as a whole.

The importance of focusing on this subgroup of RA patients was previously acknowledged by an international survey among rheumatologists [5]. This survey indicated that several topics that are considered important for the management of D2T RA are not addressed in current RA management recommendations, reflecting an unmet clinical need. Additionally, results showed a wide variety in the existing concepts of D2T RA. Consequently, a European League Against Rheumatism (EULAR, from 2021 European Alliance of Associations for Rheumatology) Task Force recently defined D2T RA (Supplemental table 1) [8] and specific management recommendations for this patient population are under development [8–10].

In the process of developing these recommendations, it became clear that evidence regarding this patient population is scarce and that further research is urgently needed [9, 10]. This is however complicated by the difficulty of identifying D2T RA patients both retrospectively in cohorts and prospectively in clinical practice, due to the multidimensionality of the D2T RA definition and the presumed fluctuation of the disease state over time. Additionally, D2T RA comprises a heterogeneous group of patients with potential differences in contributing factors and underlying pathology [6, 8, 11]. Identifying D2T RA patients in routine care data enhances research opportunities, as it allows to retrospectively study the development of RA into D2T RA and the progression of the D2T RA state over time. Clear identification of these patients in retrospective data could also enable the development of models that can predict the development of D2T RA early on in the disease course, ultimately aiding in preventing D2T RA by a timely adjustment of therapy.

We previously conducted a cross-sectional study at the department of Rheumatology & Clinical Immunology of the University Medical Center Utrecht (UMC Utrecht), the Netherlands, in which RA patients meeting the D2T RA definition [8] and a control group of RA patients not fulfilling all three criteria of the definition were enrolled [6]. This resulted in a valuable dataset with elaborate information on clinically classified D2T and non-D2T RA patients. This data served as a validation set during a hackathon (November 2020), in which data scientists and clinicians collaborated to identify and predict the development of D2T RA in structured and unstructured routine care data of all RA patients at UMC Utrecht.

## Methods

### Routine care data

Structured and unstructured routine care data were extracted from the Utrecht Patient Oriented Database (UPOD) and pseudonymized. The organization and content of the UPOD have been described in more detail elsewhere [12]. In brief, the UPOD is an infrastructure of relational databases comprising electronic health record data of all patients treated at UMC Utrecht and was established in 2004. UPOD data acquisition and management are in accordance with current regulations concerning privacy and ethics. For this hackathon, first, we identified the RA population according to the 10th revision of International Classification of Diseases (ICD-10) codes. We included patients with classification M05.X (seropositive rheumatoid arthritis) and M06.X (other rheumatoid arthritis) and subsequently excluded patients with M06.1 (adult-onset Still disease). Subsequently, the following structured data were extracted from the UPOD:

- Age (at time of RA diagnosis) and sex
- Medication prescriptions: We included relevant medication based on Anatomical Therapeutic Chemical (ATC) codes (Supplemental table 2). All inpatient and outpatient prescriptions, including ATC codes and start dates, were extracted. As medication stop dates are prone to administrative errors, we only used start dates in our analyses. The b/tsDMARDs were labeled according to their mechanism of action (MoA). Medication prescriptions dated back to 2007.
- Laboratory analyses: We extracted laboratory measurements deemed clinically relevant (Supplemental table 3). In addition, we included all hematological parameters, as these are available in the UPOD for all patients for whom one or more components of the complete blood count (CBC) have been requested (e.g., hemoglobin). These parameters include the entire CBC, as well as research-only values and raw scatter pattern measurements from the Abbott Celldyn Sapphire machines (Abbott hematology, Santa Clara, CA, USA). This data was available from 2003.
- Clinical measurements: Clinical measurements including 28 joint counts for swelling and for tenderness (SJC28/TJC28), length, weight, blood pressure, and general health related to RA according to the patient as scored on a visual analog scale (VAS-GH) were extracted for all patients. This data was available since 2002.
- Hospital visits: Visits to the outpatient rheumatology clinic (since 1995) as well as hospitalizations on the rheumatology ward (since 1987) were extracted for all patients.

In addition, clinical correspondence was extracted as unstructured data from the UPOD. This included all clinical letters from the rheumatology department as available since 1988.

### Clinically classified patients

In a previous cross-sectional study [6], 52 D2T and 100 non-D2T RA patients were clinically classified according to the EULAR definition (Supplemental table 1) in 2019–2020 [8]. See Supplemental table 4 for an overview of the clinical characteristics of these patients. Both the structured and unstructured UPOD data as well as the study data were extracted. Study data included patient and disease characteristics as well as factors potentially contributing to D2T RA (e.g., treatment non-adherence, fibromyalgia), which were collected during a single study visit including a physical examination, laboratory analyses, and by a subsequent questionnaire set. The data from these clinically classified patients served as a validation set, used to define the ability of the identification and prediction models to correctly classify D2T RA patients.

### Identification of D2T RA patients

Four different techniques were employed to identify D2T RA patients in routine care data. The first two were based on the application of the criteria of the D2T RA definition in structured and unstructured data, respectively. Both methods focused on the first two criteria of the D2T RA definition (failing ≥ 2 b/tsDMARDS with different MoA and signs of active/progressive disease, see Supplemental table 1 for details) [8]. The third criterion (problematic management) was deemed too subjective to be extracted from the available data. The third method explored the ability of other variables available in the structured data to differentiate D2T from non-D2T RA patients using a feature importance analysis. The fourth method entailed an exploratory dimension reduction of longitudinal hematological data.

#### Classification in structured data

In this approach, the structured data of medication prescriptions, laboratory analyses, clinical measurements, diagnostic codes, and hospital visits were analyzed for all RA patients in the UPOD. Patients were classified as D2T or non-D2T RA using these data (Supplemental table 1) [8]. Patients with registered medication prescriptions of at least two b/tsDMARDs with different MoA were deemed eligible to meet the first criterion of the D2T RA definition [8]. To define “active disease” (second criterion), we aimed to calculate the disease activity score assessing 28 joints (DAS28) from SJC28/TJC28 and VAS-GH combined with erythrocyte sedimentation rate (ESR) or C-reactive protein (CRP) where available. However, as these were missing for many patient visits in the database, a model was developed that approximated the DAS28-ESR. This model was based on laboratory values, number of hospital visits, patient characteristics and swiftness of cycling through b/tsDMARDs with a different MoA (see Supplemental table 5 for a brief description of the model and an overview of included parameters). The model had a mean absolute error of 0.8 (for reference: the DAS28 itself has a measurement error of 0.6) [13]. Patients who had a mean approximated DAS28-ESR ≥ 3.2 in the period from 3 to 12 months after starting a b/tsDMARD of a second MoA were deemed to have failed their treatment due to active disease, thus fulfilled the first and second criterion of the D2T RA definition [8]. Patients who started a third b/tsDMARD with a different MoA were also deemed to have failed the b/tsDMARD of a second MoA and thus also met the first and second criterion of the D2T RA definition. This way, the RA patients in the UPOD dataset could be classified as being either D2T or non-D2T based on the available structured data.

#### Classification in unstructured data

In this approach, text mining techniques were applied to analyze clinical letters of RA patients in the UPOD to classify patients as D2T or non-D2T RA (Supplemental table 1) [8]. Medication prescriptions were extracted from the headings “medication” and “DMARD history”. Patients who had a history of a prescription of at least 2 b/tsDMARDs with different MoA were deemed to meet the first criterion of the D2T RA definition. To meet the second criterion, relevant subheadings were screened for synonyms of active disease, such as “flare”. Negations such as “no flare” were excluded. This way, the RA patients in the UPOD dataset could be classified as being either D2T or non-D2T based on the available unstructured data.

#### Feature importance analysis

To gain insight in the importance of structured data variables regarding their ability to differentiate D2T from non-D2T RA patients, we performed an exploratory feature importance analysis using logistic regression. We included all available structured data variables from the UPOD of the 152 clinically classified patients, including those used for the application of the EULAR definition [8]. We determined the importance of different variables with multivariable logistic regression with L1 regularization (based on 1000 bootstrapped cross-validations with a 140/12 split). L1 regularization limits the number of coefficients by eliminating uninformative coefficients. This was preceded by standard scaling and multiple imputation using Bayesian Ridge regression and univariate feature filtering using a false discovery rate with alpha 0.05. The repeated measured variables were time-aggregated using the mean, median, standard deviation, mean difference, and mean minus the median. The resulting variables were univariately filtered based on their ability to differentiate between D2T and non-D2T RA patients. An identification model was derived using XGBoost, of which we present the receiver operating characteristic (ROC) curve based on ten-fold cross-validation. XGBoost is a machine learning model which uses gradient boosting [14]. In gradient boosting, multiple decision tree models are combined together into an ensemble. Each sequential model is trained to correct for the errors of the previous model. An important advantage of XGBoost is that it can handle missing data without imputation, which makes it a suitable model for real-life EHR data. We also considered multivariate logistic regression and a dense neural network, but the XGBoost model had a better performance in terms of AUC.

#### Dimension reduction of longitudinal hematological data

To explore the possibility to differentiate D2T from non D2T RA patients solely based on longitudinal hematological data, a non-linear dimensionality reduction was performed. In dimension reduction, all available hematological parameters are reduced to two parameters, which allows for this information to be plotted on a 2-dimensional x-y graph. Dimension reduction was performed using uniform manifold approximation and projection (UMAP) [15]. UMAP is a non-linear alternative to principal component analysis, which explicitly aims to preserve the Euclidean distance between samples.

This method was applied to all hematological data of the 152 clinically classified patients for training purposes using supervised techniques. Subsequently, this method was applied to the hematological data of all RA patients from the UPOD, to assess its ability to differentiate D2T from non-D2T RA patients. A Y-score was calculated for each patient, indicating the likelihood of having D2T RA. This was based on the combined outcomes of the classifications in structured and unstructured data (as described above), and the clinical classification (if available).

The results of these analyses are visualized for each individual patient using the median of the reduced dimensions (d1 and d2) of the hematological data over time. This was done both for the clinically classified patients as well as all RA patients from the UPOD. The aim of this method is to investigate if distinct clusters can be distinguished to separate D2T from non-D2T RA patients based on longitudinal hematological data.

### Prediction model

In an effort to predict D2T RA patients early in the disease course (i.e., before satisfying the D2T RA definition), we developed a prediction model based on machine learning techniques using XGBoost [14]. All available structured UPOD data from before the start of the first b/tsDMARD of the clinically classified D2T and non-D2T RA patients were used. The longitudinal data were regularized to a one-month time interval using forward fill-in. This implies that missing values are imputed based on the last known values. The XGBoost classifier was used as the predictive model because of its robustness regarding data preprocessing. We used 10-fold cross-validation and the area under the ROC (AUC) statistic to determine model performance.

## Results

### Data extraction from the UPOD

Based on the ICD-10 codes, 1873 RA patients were identified in the UPOD.

### Identification

#### Classification in structured data

Of the 1873 RA patients in the UPOD, 122 patients met the first criterion of the D2T RA definition (7%) as determined in structured UPOD data. For 100 of these patients, sufficient data was available to determine the fulfilment of the second criterion. Patients for whom insufficient data was available were classified as non-D2T. Twenty-five of 52 patients clinically classified as D2T RA patients were correctly classified based on the structured data (sensitivity 48%, see Table 1). Two of the 100 patients clinically classified as non-D2T RA were incorrectly classified (specificity 98%, Table 1). Using this approach, 43 additional (potential) D2T RA patients were identified.

**Table 1:** Classification of D2T and non-D2T patients in structured routine care data

Classification in structured data	Validation			
	Clinically classified D2T RA*	Clinically classified non-D2T RA*	Newly classified patients in the UPOD	Total
D2T RA	25	2	43	70
Non-D2T RA	27	98	1678	1803
Total	52	100	1721	1873

Patients were classified by applying the D2T RA definition [8] in structured routine care data from the UPOD

*D2T* difficult-to-treat, *DAS28-ESR* disease activity score based on 28-joint count and erythrocyte sedimentation rate, *RA* rheumatoid arthritis, *UPOD* Utrecht Patient Oriented Database

*Clinical classification of D2T and non-D2T RA patients as performed in the cross-sectional study [6]

#### Classification in unstructured data

In the UPOD, 16,780 clinical letters of 1873 patients were available and extracted as unstructured data. Two-hundred thirty-nine of all RA patients from the UPOD (13%) met the first D2T RA criterion, based on the unstructured data. This included all 52 clinically classified D2T RA patients from the cross-sectional study. One hundred sixty-one patients also met the second criterion of the definition. Thirty-six of 52 patients clinically classified as D2T RA patients were correctly classified using the unstructured data (sensitivity 69%, see Table 2). Eight of the 100 patients clinically classified as non-D2T RA were incorrectly classified (specificity 92%, Table 2). One hundred and seventeen additional (potential) D2T RA patients were identified. When comparing these patients with the 43 identified additional (potential) D2T RA patients using the structured data approach, 123 unique, additional (potential) D2T RA patients were found.

**Table 2:** Classification of D2T and non-D2T patients in unstructured routine care data

Classification in unstructured data	Validation			
	Clinically classified D2T RA*	Clinically classified non-D2T RA*	Newly classified patients in the UPOD	Total
D2T RA	36	8	117	161
Non-D2T RA	16	92	1604	1712
Total	52	100	1721	1873

Patients were classified by applying the D2T RA definition [8] in unstructured routine care data from the UPOD

*D2T* difficult-to-treat, *RA* rheumatoid arthritis, *UPOD* Utrecht Patient Oriented Database

*Clinical classification of D2T and non-D2T RA patients as performed in the cross-sectional study [6]

#### Feature importance analysis

**Fig. 1:**
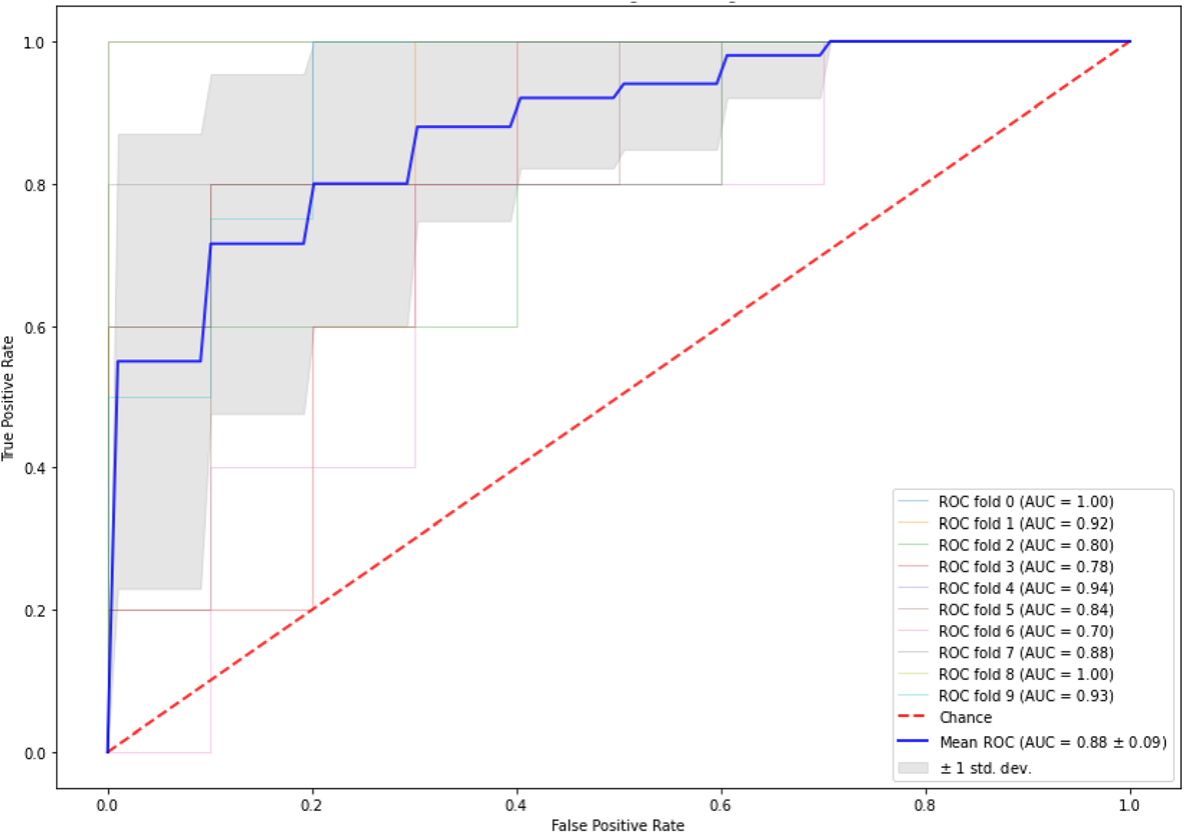
ROC-curve of the D2T RA identification model based on a feature importance analysis. AUC-ROC for an identification model to identify D2T and non-D2T RA patients based on structured UPOD data. The model is based on the most important features derived with logistic regression techniques from the available structured data from the UPOD. D2T, difficult-to-treat; RA, rheumatoid arthritis; AUC, area under the curve; ROC, receiver-operator curve; UPOD, Utrecht Patient Oriented Database

The most important structured data variables (features) to identify D2T and non-D2T RA patients and their logistic regression coefficients are shown in Tables 3 and 4. Among others, this included the number of different medication prescriptions, the time period since RA diagnosis, and the mean DAS28-ESR. Based on these features, an identification model was derived with an AUC-ROC of 0.88 (95% CI 0.82–0.94), Fig. 1.

**Table 3:** The most important features to identify D2T RA patients based on logistic regression coefficients

Feature	Logistic regression coefficient
Number of different medication prescriptions, based on the extracted medication in Supplemental table 2	1.05
Mean DAS28-ESR score over time	0.76
Median DAS28-ESR score over time	0.70
Median non-invasively measured blood pressure over time	0.64
Standard deviation of the creatinine laboratory measurements over time	0.63
Time since RA diagnosis	0.52
Median of banded neutrophils over time	0.37
Ratio of segmented neutrophils by percentage of immature granulocytes over time	0.30
Standard deviation of percentage of reticulocytes over time	0.30
Median of the delta over time of banded neutrophils over time	0.29

Features are noted in order of importance. A higher value of a feature corresponds to a higher likelihood of having D2T RA

*DAS28* disease activity score based on 28-joint count, *ESR* erythrocyte sedimentation rate, *RA* rheumatoid arthritis

**Table 4:** The most important features to identify non-D2T RA patients based on logistic regression coefficients

Feature	Logistic regression coefficient
Maximum ESR over time	0.84
Standard deviation of ESR values over time	0.78
Mean minus median of intermediate angle scatter of platelets over time	0.63
White blood cell count divided by lymphocyte concentration over time	0.62
Median length	0.58
Minimum potassium value over time	0.56
Female sex	0.56
Median neutrophils over time	0.46
Median percentage of reticulocytes over time	0.43
Standard deviation of DAS28-ESR score over time	0.43

Features are noted in order of importance. A higher value of a feature corresponds to a higher likelihood of having non-D2T RA

*DAS28* disease activity score based on 28-joint count, *ESR* erythrocyte sedimentation rate, *IAS* intermediate angle scatter of platelet

#### Dimension reduction of longitudinal hematological data

**Fig. 2:**
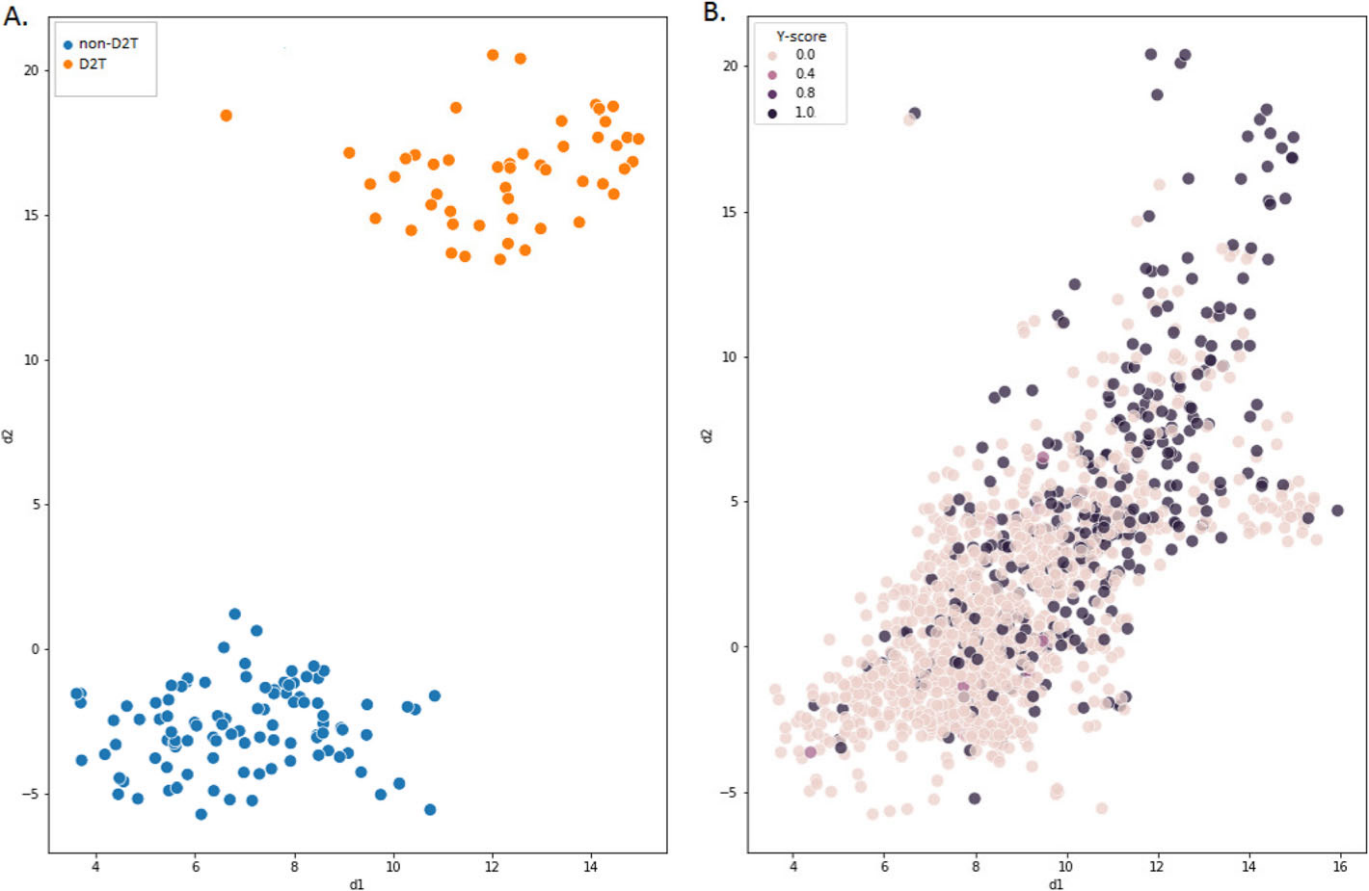
Reduced dimensions of longitudinal hematological data. **A** Medians of the reduced dimensions of the longitudinal hematological data of all 52 clinically classified D2T and 100 clinically classified non-D2T RA patients. **B** Medians of the reduced dimensions of the longitudinal hematological data of all 1873 RA patients in the UPOD-database, where a higher Y-score indicates a higher estimated probability of having D2T RA according to the classifications in structured and unstructured data, and the clinical classification (if available). All available hematological parameters were reduced to two dimensions (d1 and d2). For each patient, the median of these reduced dimensions over time is visualized. d, reduced dimension; D2T, difficult-to-treat; RA, rheumatoid arthritis; UPOD, Utrecht Patient Oriented Database

Figure 2A depicts the medians of the reduced dimensions of the longitudinal hematological data of the clinically classified D2T and non-D2T RA patients. Each point represents a single patient, and the axes represent the two reduced dimensions d1 and d2. Two distinct clusters are visible, which are strictly separated due to the supervised techniques. Figure 2B depicts the medians of the reduced dimensions of the hematological data of all 1873 RA patients in the UPOD. A tendency towards two separate clusters is visible based on the likelihood of having D2T RA, although these are not strictly separated.

### Prediction model

**Fig. 3:**
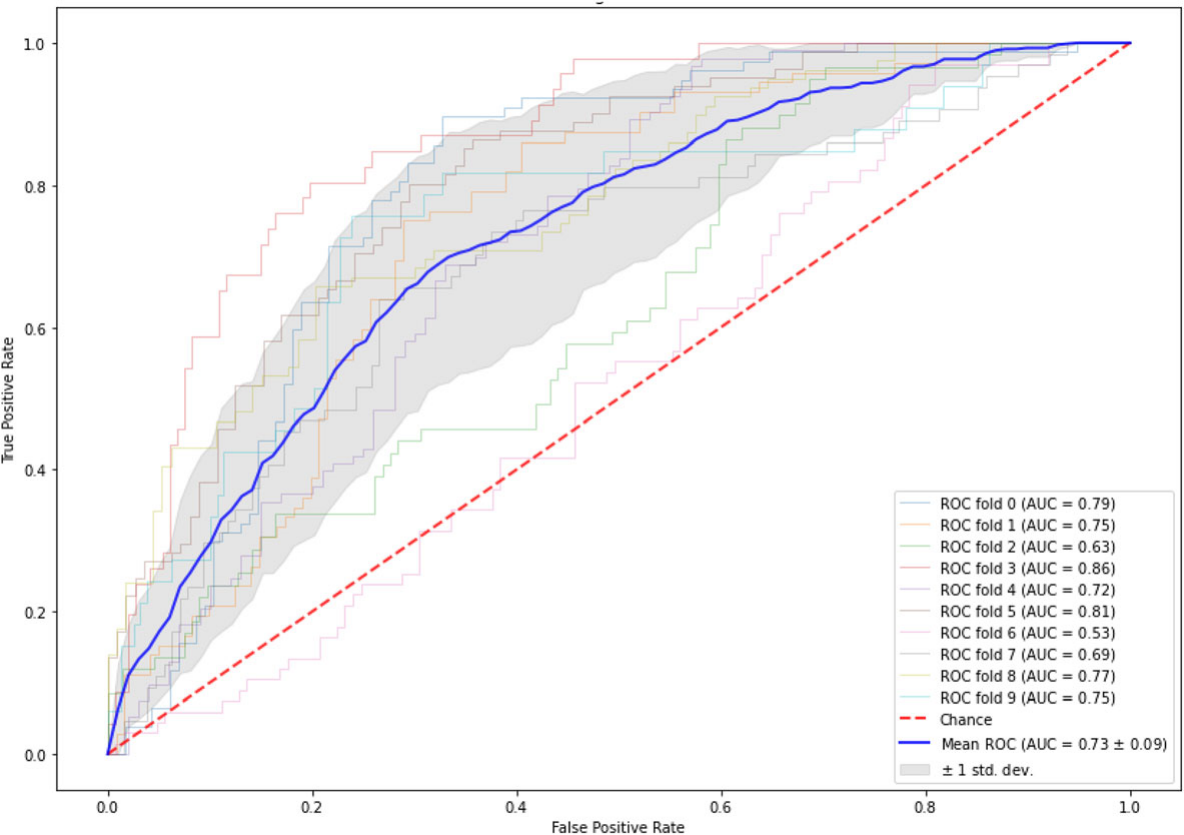
ROC-curve of the D2T RA machine learning prediction model. AUC-ROC of the D2T RA prediction model based on data from before the start of the first b/tsDMARD. AUC, area under the curve; b/tsDMARD, biological or targeted synthetic disease-modifying antirheumatic drug; csDMARD, conventional synthetic disease-modifying antirheumatic drug; D2T, difficult-to-treat; RA, rheumatoid arthritis; ROC, receiver-operator characteristic; std dev, standard deviation

The machine learning prediction model was trained on the data of the clinically classified RA patients for whom data was available before prescribing the first b/tsDMARD (28 D2T and 88 non-D2T RA patients). The most important features mainly included hematological parameters, e.g., white blood cell count, percentage of neutrophils, segmented neutrophils, and hemoglobin (see Supplemental Table 6 for further details). With this XGBoost model, we were able to correctly predict 22 of the clinically classified D2T RA patients and 44 of the clinically classified non-D2T RA patients (sensitivity 79%, specificity 50%, Table 5). The average AUC-ROC over the 10-fold cross-validation was 0.73 (95% CI 0.71–0.75), Fig. 3.

**Table 5:** The number of predicted D2T and non-D2T RA patients

Prediction	Validation		
	Clinically classified D2T RA	Clinically classified non-D2T RA	Total
D2T RA	22	44	66
Non-D2T RA	6	44	50
Total	28	88	116

Predictions are based on data from before the start of the first b/tsDMARD

*b/tsDMARD* biological or targeted synthetic disease-modifying antirheumatic drug, *D2T* difficult-to-treat, *RA* rheumatoid arthritis

*Clinical classification of D2T and non-D2T RA patients as performed in the cross-sectional study [6]

A decision threshold of 0.15 was applied

## Discussion

The current study presents the results of a hackathon aimed at the identification and prediction of D2T RA patients in structured and unstructured routine care data. We were able to identify 123 potentially new D2T RA patients by applying the criteria of the D2T RA definition in structured and unstructured data. Additionally, we developed an identification model based on a feature importance analysis with high diagnostic performance (AUC-ROC 0.88), and we have shown the potential of longitudinal hematological parameters to differentiate D2T from non-D2T RA patients using supervised dimension reduction. To predict the risk of developing D2T RA, we developed a machine learning model based on structured data that correctly predicted 79% of clinically classified D2T RA patients using data available from before the time of prescribing the first b/tsDMARD (AUC-ROC 0.73). To our knowledge, there is no previous literature using these techniques in the context of (D2T) RA.

Routine care data is a valuable source of information, as it comprises a vast amount of “real world” patient data that is ample available. Unfortunately, this data often remains unutilized, due to technical challenges in their analysis. Yet routine care data could play a crucial role in the developing field of personalized medicine. A major strength of this study is that we have shown various data analytical techniques to utilize this valuable source of information in the identification and prediction of D2T RA. Identifying D2T RA patients from routine care data enhances research possibilities, as it allows for retrospective analysis of the development of RA into D2T RA and the progression of the D2T RA state over time. Moreover, in clinical practice, it creates an opportunity to optimize the treatment of D2T RA patients according to current and emerging guidelines. Correct identification of patients in longitudinal routine care data may also enhance the performance of models that can predict D2T RA early in the disease course. When patients at risk can be identified at an early stage, they may be monitored more intensively for the presence or development of factors contributing to D2T RA (e.g., treatment non-adherence or depression) [6]. When these contributing factors develop and are adequately addressed, the risk of acquiring D2T RA could potentially be diminished.

Interestingly, our feature importance analysis, our machine learning prediction model, and our exploratory dimension reduction all show an important role for hematological data in the identification and prediction of D2T RA patients. This is in line with previous research that has shown the potential role of the neutrophil-lymphocyte and platelet-lymphocyte ratios as biomarkers of disease activity in RA patients, although the underlying pathophysiology is not well-understood [16–18]. Of note, the large contribution of hematological parameters in our analyses is likely influenced by the ample availability of these structured data, as this is a key feature of the UPOD. Nevertheless, as hematological parameters are low in costs, often readily available, and require a minimal effort of the treating physician, they could be valuable potential markers in the evaluation of RA disease progression.

The performance of our identification strategies based on structured and unstructured data has been estimated conservatively. Patients for whom insufficient data were available to apply the D2T RA definition were now classified as “non-D2T”, which may have contributed to the relatively low sensitivity that was observed. The D2T RA patients that were not identified by our models could especially include the D2T RA patients who were referred to UMC Utrecht from other hospitals as a “second opinion”, as data transfers between hospitals are often incomplete and electronic health record data from different hospitals, general practitioners, and pharmacies are (unfortunately) not synchronized in the Netherlands. Improving the availability of these data could thus potentially improve the performance of our identification and, subsequently, prediction models.

Although the results of this study are promising regarding the accuracy of identification of D2T RA patients as well as predicting the development of D2T RA, this preliminary study also has several limitations. For example, not all components of the D2T RA definition (Supplemental table 1) [8] were incorporated in the structured and unstructured data approaches. This was done for several reasons. First of all, the subjective character of criterion 3 “the management of the signs and/or symptoms is perceived as problematic by the rheumatologist and/or the patient” was deemed too subjective to extract from the available data. Additionally, whether the management of patients is perceived as problematic will most often not be routinely noted in health records. This issue will therefore remain a challenge in further research on D2T RA. Second, for criterion 2c “inability to taper glucocorticoid treatment below 7.5mg/day prednisone or equivalent”, the stop dates of the medication that are available in the digital prescriptions system were deemed too unreliable. For example, additional medication prescriptions may be requested from the general practitioner instead of the rheumatologist (which are noted in separate systems), resulting in missing data in the prescription system and incorrect stop dates. Inclusion of these criteria in future identification and/or prediction models could further improve their performance. Furthermore, an inherent limitation of working with routine care data is the dependency on the availability of certain data parameters. Several factors that have previously been reported in association with more severe RA disease activity, such as smoking status and radiographic progression, were not readily available in the UPOD [19, 20]. Improvement of registration of these parameters and the optimization of free text mining techniques could allow for future inclusion of these parameters in model development resulting in still better performing prediction models.

In future studies, the possibility of combining the different techniques presented in this paper for the identification of D2T RA patients in structured and unstructured routine care data should be addressed. In addition, other data sources could be utilized to explore other known contributing and risk factors for D2T RA, such a low socio-economic status based on, e.g., postal codes [6, 21]. Furthermore, the performance of the presented identification and prediction models should be evaluated in external data.

## Conclusions

In conclusion, during this hackathon, we have demonstrated potential techniques (including text mining, feature importance analysis, and machine learning) for the identification and prediction of D2T RA patients in structured and unstructured routine care data. The results are promising to fuel research in this emerging field and should be optimized in further research.

## Supplementary Information

**Additional file 1: Supplemental table 1.** EULAR definition of D2T R A[8]. **Supplemental table 2.** Selected medication and ATC codes for extraction from the Utrecht Patient Oriented Database (UPOD). **Supplemental table 3.** Selected laboratory measurements for extraction from the Utrecht Patient Oriented Database (UPOD). **Supplemental table 4.** Patient characteristics of clinically classified D2T and non-D2T patients. **Supplemental table 5.** Most important features of machine learning model to predict the DAS28-ESR*. **Supplemental table 6.** Most important features of the machine learning model to predict the development of D2T RA before the start of the first b/tsDMARD.

## Data Availability

Due to the nature of this research, patients in this study did not agree for their data to be shared publicly, so supporting data is not available.

## Abbreviations

ATC: Anatomical Therapeutic Chemical
AUC: Area under the ROC
(b/ts)DMARDs: (Biological and/or targeted synthetic) disease-modifying antirheumatic drugs
CBC: Complete blood count
CI: Confidence interval
CRP: C-reactive protein
D2T: Difficult-to-treat
DAS28: Disease activity score assessing 28 joints
ESR: Erythrocyte sedimentation rate
EULAR: European League Against Rheumatism (from 2021 European Alliance of Associations for Rheumatology)
ICD-10: International Classification of Diseases
MoA: Mechanism of action
RA: Rheumatoid arthritis
ROC: Receiver operating characteristic
SJC28: 28 Joint count for swelling
TJC28: 28 Joint count for tenderness
UMAP: Uniform manifold approximation and projection
UMC: University Medical Center
UPOD: Utrecht Patient Oriented Database
VAS-GH: General health related to RA according to the patient as scored on a visual analog scale
