# Supplementary material for: Identification and prediction of difficult-to-treat rheumatoid arthritis patients in structured and unstructured routine care data: results from a hackathon
Source: Arthritis Res Ther. 2021 Jul 8;23:184. doi: 10.1186/s13075-021-02560-5 (PMC8265126; doi:10.1186/s13075-021-02560-5)
Supplement: Supplementary file 1 — Additional file 1: Supplemental table 1. EULAR definition of D2T R A[8]. Supplemental table 2. Selected medication and ATC codes for extraction from the Utrecht Patient Oriented Database (UPOD). Supplemental table 3. Selected laboratory measurements for extraction from the Utrecht Patient Oriented Database (UPOD). Supplemental table 4. Patient characteristics of clinically classified D2T and non-D2T patients. Supplemental table 5. Most important features of machine learning model to predict the DAS28-ESR*. Supplemental table 6. Most important features of the machine learning model to predict the development of D2T RA before the start of the first b/tsDMARD. [file 13075_2021_2560_MOESM1_ESM.docx]

**Supplemental table 1.** EULAR definition of D2T RA[8]

| 1. Treatment according to EULAR recommendations and failure of ≥2 b/tsDMARDs (with different mechanisms of action)^1^ after failing csDMARD therapy (unless contraindicated)^2^ 2. Signs suggestive of active/progressive disease, defined as ≥1 of:    1. At least moderate disease activity (according to validated composite measures including joint counts e.g. DAS28-ESR > 3.2 or CDAI >10)    2. Signs (including acute phase reactants and imaging) and/or symptoms suggestive of active disease (joint related or other)    3. Inability to taper glucocorticoid treatment (below 7.5 mg/day prednisone or equivalent)    4. Rapid radiographic progression (with or without signs of active disease)^3^    5. Well-controlled disease according to above standards, but still having RA symptoms that are causing a reduction in quality of life 3. The management of signs and/or symptoms is perceived as problematic by the rheumatologist and/or the patient |
| --- |

All three criteria need to be present in D2T RA.

b: biological; CDAI: clinical disease activity index; cs: conventional synthetic; DAS28-ESR: disease activity score assessing 28 joints using erythrocyte sedimentation rate; DMARD: disease-modifying antirheumatic drug; EULAR: European League Against Rheumatism, from 2021 European Alliance of Associations for Rheumatology; mg: milligram; RA: rheumatoid arthritis; ts: targeted synthetic.

1) Unless restricted by access to treatment due to socioeconomic factors.

2) If csDMARD treatment is contraindicated, failure of ≥2 b/tsDMARDs with different mechanisms of action is sufficient.

3) Rapid radiographic progression: change in van der Heijde-modified Sharp score ≥ 5 points at 1 year[21]

**Supplemental table 2.** Selected medication and ATC codes for extraction from the Utrecht Patient Oriented Database (UPOD)

| Category | ATC code | Description |
| --- | --- | --- |
| Glucocorticoid | H02AB01 | Betamethasone |
| Glucocorticoid | H02AB02 | Dexamethasone |
| Glucocorticoid | H02AB04 | Methylprednisolone |
| Glucocorticoid | H02AB06 | Prednisolone |
| Glucocorticoid | H02AB07 | Prednisone |
| Glucocorticoid | H02AB08 | Triamcinolon |
| Glucocorticoid | H02AB09 | Hydrocortison |
| csDMARD | A07EC01 | Sulfasalazine |
| csDMARD | L01AA01 | Cyclophosphamide |
| csDMARD | L01BA01 / L04AX03 | Methotrexate |
| csDMARD | L04AX01 | Azathioprine |
| csDMARD | L04AA13 | Leflunomide |
| csDMARD | L04AD01 | Ciclosporin |
| csDMARD | P01BA01 | Chloroquine |
| csDMARD | P01BA02 | Hydroxychloroquine |
| bDMARD – CD20 inhibitor | L01XC02 | Rituximab |
| bDMARD - TNFi | L04AB01 | Etanercept |
| bDMARD – TNFi | L04AB02 | Infliximab |
| bDMARD – TNFi | L04AB04 | Adalimumab |
| bDMARD – TNFi | L04AB05 | Certolizumab pegol |
| bDMARD – TNFi | L04AB06 | Golimumab |
| bDMARD – CTLA4 inhibitor | L04AA24 | Abatacept |
| bDMARD – IL-1 inhibitor | L04AC03 | Anakinra |
| bDMARD – IL-6 receptor antagonist | L04AC07 | Tocilizumab |
| bDMARD – IL-6 inhibitor | L04AC14 | Sarilumab |
| tsDMARD – JAK inhibitor | L04AA29 | Tofacitinib |
| tsDMARD – JAK inhibitor | L04AA37 | Baricitinib |

ATC: Anatomical Therapeutic Chemical; b: biological; cs: conventional synthetic; DMARD: disease-modifying antirheumatic drug; IL: interleukin; JAK: janus kinase; TNFi: tumor necrosis factor inhibitor; ts: targeted synthetic.

**Supplemental table 3.** Selected laboratory measurements for extraction from the Utrecht Patient Oriented Database (UPOD)

| 25-OH-Vitamin D | Low-density lipoprotein cholesterol |
| --- | --- |
| Anti-citrullinated protein antibodies | Lactate dehydrogenase |
| Albumin | Lipase |
| Alanine transaminase | M-protein screening |
| Alkaline phosphatase | Magnesium |
| Amylase | P-antineutrophil cytoplasmic antibodies |
| Antinuclear antibodies | Parathyroid hormone |
| Activated partial thromboplastin time | Phosphate |
| Aspartate transaminase | Potassium |
| Bicarbonate | Prothrombin time |
| Total bilirubin | Prothrombin time and international normalized ratio |
| Brain natriuretic peptide | Rheumatoid factor |
| C-antineutrophil cytoplasmic antibodies | Sodium |
| CA 15.3 | Soluble interleukin-2 receptor |
| Calcium | Total protein level |
| Cholesterol | Transferrin |
| Creatinine kinase | Transferrin saturation |
| Creatinine kinase-myocardial band mass | Triglycerides |
| Creatinine | Troponin |
| CRP | Thyroid-stimulating hormone |
| Erythrocyte sedimentation rate | Uric acid |
| Ferritin | Vitamin B1 |
| Folic acid | Vitamin B12 |
| Free T4 | Vitamin B6 |
| GGT | Sediment (urine) |
| Glucose | Creatinine (urine) |
| Hemoglobulin A1c | Uric acid (urine) |
| High-density lipoprotein cholesterol | Creatinine (collected urine) |
| Immunoglobulin G total | Uric acid (collected urine) |
| Iron |  |

**Supplemental table 4.** Patient characteristics of clinically classified D2T and non-D2T patients

|  | Clinically classified  D2T RA (n=52) | Clinically classified  non-D2T RA (n=100) |
| --- | --- | --- |
| Age (mean, SD) | 60.2 (11.4) | 64.5 (10.9) |
| Female, % | 73 | 72 |
| Disease duration, years (median, IQR) | 17.0 (9.0-25.0) | 14.0 (8.0-24.0) |
| Age at onset, years (mean, SD) | 41.9 (12.4) | 47.8 (15.6) |
| RF positivity, % | 75 | 65 |
| ACPA positivity, % | 73 | 65 |
| Joint erosions, % | 63 | 49 |
| DAS28-ESR (median, IQR) | 4.1 (3.5-6.1) | 2.5 (1.8-3.3) |
| csDMARD(s), % | 71 | 86 |
| bDMARD, % | 52 | 39 |
| tsDMARD, % | 23 | 0 |

Data is based on the cross-sectional study of Roodenrijs et al.[6]
ACPA: anti-citrullinated protein antibody; b: biological; cs: conventional synthetic; DAS28: disease activity score based on 28 joint count; DMARD: disease modifying anti-rheumatic drug; D2T: difficult-to-treat; ESR: erythrocyte sedimentation rate; IQR: interquartile range; RF: rheumatoid factor; SD: standard deviation; ts: targeted synthetic.

**Supplemental table 5.** Most important features of machine learning model to predict the DAS28-ESR*

| Feature | Shapley value (order of importance) | Direction of the effect |
| --- | --- | --- |
| Minimum hemoglobin over time | 0.151 | - |
| Higher age | 0.127 | - |
| Maximum platelet count as measured by impedance over time | 0.100 | + |
| Maximum fraction of immature reticulocytes over time | 0.078 | - |
| Minimum platelet crit over time | 0.063 | - |
| Female gender | 0.063 | - |
| Maximum platelet crit over time | 0.056 | + |
| Mean size of neutrophils over time, measured by the mean axial light loss | 0.055 | + |
| Number of visits to the outpatient clinic in the past 6 months | 0.054 | + |
| Maximum red cell distribution width over time | 0.053 | + |

DAS28: disease activity score based on 28-joint count; ESR: erythrocyte sedimentation rate

*:The DAS28-ESR prediction model was trained on all available DAS28-ESR scores in the Utrecht Patient Oriented Database. The model was developed using the machine learning model XGBoost,[22] which uses gradient boosting. In gradient boosting, multiple decision tree models are combined together into an ensemble. Each sequential model is trained to correct for the errors of the previous model. An important advantage of XGBoost is that it can handle missing data without imputation, which makes it a suitable model for real-life EHR data. Feature importance was determined with Shapley values, which determines the contribution of a feature to each tree in the model. Note that this method is non-linear and there are feature interactions. The third column indicates the direction of the effect on the outcome variable. The DAS28-ESR prediction model had a mean absolute error of 0.8.

**Supplemental table 6.** Most important features of the machine learning model to predict the development of D2T RA before the start of the first b/tsDMARD

| Feature | Shapley value (order of importance) | Direction of the effect |
| --- | --- | --- |
| Percentage macrocytic erythrocytes, difference between maximum and minimum values over time | 1.016 | + |
| White blood cell count, difference between maximum and minimum values over time | 0.664 | + |
| Variance of polarized side scatter of platelets, difference between maximum and minimum values over time | 0.620 | - |
| Minimum non-invasively measured blood pressure over time | 0.568 | + |
| White cell viability, difference between maximum and minimum values over time | 0.511 | + |
| Minimum weight of patient | 0.495 | + |
| Mean polarized side scatter of neutrophils, difference between maximum and minimum values over time | 0.411 | + |
| Hemoglobin, difference between maximum and minimum values over time | 0.410 | + |
| Percentage of neutrophils, difference between maximum and minimum values over time | 0.379 | + |
| Segmented neutrophils, difference between maximum and minimum values over time | 0.378 | + |

b/tsDMARD: biological or targeted synthetic disease-modifying antirheumatic drug; D2T: difficult-to-treat; RA: rheumatoid arthritis.
The model was developed using the machine learning model XGBoost,[22] which uses gradient boosting. In gradient boosting, multiple decision tree models are combined together into an ensemble. Each sequential model is trained to correct for the errors of the previous model. An important advantage of XGBoost is that it can handle missing data without imputation, which makes it a suitable model for real-life EHR data. The order of importance was determined with Shapley values, which determines the contribution of a feature to each tree in the model. Note that this method is non-linear and there are feature interactions. The third column indicates the direction of the effect on the outcome variable.
